# Supplementary material for: HIVprotI: an integrated web based platform for prediction and design of HIV proteins inhibitors
Source: J Cheminform. 2018 Mar 9;10:12. doi: 10.1186/s13321-018-0266-y (PMC5845081; doi:10.1186/s13321-018-0266-y)
Supplement: Supplementary file 2 — Additional file 2. Source code of HIVProtI web server. [file 13321_2018_266_MOESM2_ESM.zip › HIVprotI_Source-code/hivprotI/weka-3-6-6/doc/weka/core/pmml/TargetMetaInfo.html]

TargetMetaInfo


---


|  |  |  |
| --- | --- | --- |
| **Overview** \| **Package** \| **Class** \| **Tree** \| **Deprecated** \| **Index** \| **Help** \| **Weka's home** | |  |
| **PREV CLASS**   NEXT CLASS | **FRAMES**    **NO FRAMES**     **All Classes** |  |
| SUMMARY: NESTED \| FIELD \| CONSTR \| METHOD | DETAIL: FIELD \| CONSTR \| METHOD |  |


---


## weka.core.pmml Class TargetMetaInfo

```
java.lang.Object
  weka.core.pmml.FieldMetaInfo
      weka.core.pmml.TargetMetaInfo
```

**All Implemented Interfaces:**: java.io.Serializable

---

``` public class TargetMetaInfo extends FieldMetaInfo implements java.io.Serializable ```

Class to encapsulate information about a Target.

**Version:**
:   $Revision 1.0 $

**Author:**
:   Mark Hall (mhall{[at]}pentaho{[dot]}com)

**See Also:**: Serialized Form

---

**Nested Class Summary**

**Nested classes/interfaces inherited from class weka.core.pmml.FieldMetaInfo**

`FieldMetaInfo.Interval, FieldMetaInfo.Optype, FieldMetaInfo.Value`


| **Method Summary** | |
| --- | --- |
| `double` | `applyMinMaxRescaleCast(double prediction)`             Apply min and max, rescaleFactor, rescaleConstant and castInteger - in that order (where defined). |
| `double` | `getDefaultValue()`             Get the default value (numeric target) |
| `Attribute` | `getFieldAsAttribute()`             Return this field as an Attribute. |
| `double` | `getPriorProbability(java.lang.String value)`             Get the prior probability for the supplied value. |
| `java.util.ArrayList<java.lang.String>` | `getValues()`             Get the values (discrete case only) for this Target. |

**Methods inherited from class weka.core.pmml.FieldMetaInfo**

`getFieldName, getOptype`

**Methods inherited from class java.lang.Object**

`equals, getClass, hashCode, notify, notifyAll, toString, wait, wait, wait`

**Method Detail**

### getPriorProbability

```
public double getPriorProbability(java.lang.String value)
                           throws java.lang.Exception
```

:   Get the prior probability for the supplied value.

    :   **Parameters:**: `value` - the value to get the probability for **Returns:**: the probability **Throws:**: `java.lang.Exception` - if there are no TargetValues defined or if the supplied value is not in the list of TargetValues

---


### getDefaultValue

```
public double getDefaultValue()
                       throws java.lang.Exception
```

:   Get the default value (numeric target)

    :   **Returns:**: the default value **Throws:**: `java.lang.Exception` - if there is no TargetValue defined

---


### getValues

```
public java.util.ArrayList<java.lang.String> getValues()
```

:   Get the values (discrete case only) for this Target. Note: the
    list may be empty if the pmml doesn't specify any values.

    :   **Returns:**: the values of this Target

---


### applyMinMaxRescaleCast

```
public double applyMinMaxRescaleCast(double prediction)
                              throws java.lang.Exception
```

:   Apply min and max, rescaleFactor, rescaleConstant and castInteger - in
    that order (where defined).

    :   **Parameters:**: `prediction` - the prediction to apply these modification to **Returns:**: the modified prediction **Throws:**: `java.lang.Exception` - if this target is not a continuous one

---


### getFieldAsAttribute

```
public Attribute getFieldAsAttribute()
```

:   Return this field as an Attribute.

    :   **Specified by:**: `getFieldAsAttribute` in class `FieldMetaInfo`
    :   **Returns:**: an Attribute for this field.


---


|  |  |  |
| --- | --- | --- |
| **Overview** \| **Package** \| **Class** \| **Tree** \| **Deprecated** \| **Index** \| **Help** \| **Weka's home** | |  |
| **PREV CLASS**   NEXT CLASS | **FRAMES**    **NO FRAMES**     **All Classes** |  |
| SUMMARY: NESTED \| FIELD \| CONSTR \| METHOD | DETAIL: FIELD \| CONSTR \| METHOD |  |


---
